# Supplementary material for: Polyketide synthase gene clusters in the ericoid mycorrhizal fungus Oidiodendron maius: genome-wide prediction, expression profiling, and functional characterization of knockout mutants
Source: Front Plant Sci. 2026 Sep 16;17:1924430. doi: 10.3389/fpls.2026.1924430 (PMC13623903; doi:10.3389/fpls.2026.1924430)
Supplement: Supplementary file 1 [file SupplementaryFile1.zip › Supplementary Materials/Supplementary Folder 1.zip!/Oidma1_AssemblyScaffolds_Repeatmasked/knownclusterblast/cluster56/jgi.p_Oidma1_138861_mibig_hits.html]

| MIBiG Protein | Description | MIBiG Cluster | MiBiG Product | % ID | % Coverage | BLAST Score | E-value |
| --- | --- | --- | --- | --- | --- | --- | --- |
| EPE34347.1 | Clavaminate\_synthase-like\_protein | BGC0001035 | Pneumocandin\_biosynthetic\_gene\_cluster | 40 | 95.2380952381 | 261 | 1.2e-69 |
| AMM63174.1 | AniF | BGC0001371 | Echinocandin\_B\_biosynthetic\_gene\_cluster | 40 | 96.4285714286 | 258 | 1.3e-68 |
| AFT91391.1 | HtyE | BGC0000338\_c2 | Echinocandin\_B\_biosynthetic\_gene\_cluster | 41 | 89.880952381 | 251 | 1.2e-66 |
| AGA37270.1 | 2OG-Fe(II)-oxygenase | BGC0000819 | Paraherquamide\_biosynthetic\_gene\_cluster | 39 | 97.3214285714 | 242 | 7.4e-64 |
| EPE34350.1 | Clavaminate\_synthase-like\_protein | BGC0001035 | Pneumocandin\_biosynthetic\_gene\_cluster | 39 | 91.0714285714 | 235 | 6.9e-62 |
| AFU82618.1 | 2-oxoglutarate\_dependent\_oxygenase | BGC0000998 | Guadinomine\_biosynthetic\_gene\_cluster | 31 | 58.9285714286 | 80 | 4.9e-15 |
